# Desmoplastic Melanoma: Clinical Characteristics and Survival in the US Population

**DOI:** 10.7759/cureus.4931

**Published:** 2019-06-18

**Authors:** Maria T Huayllani, Andrea Sisti, David J Restrepo, Daniel Boczar, Jordan J Cochuyt, Aaron C Spaulding, Sanjay P Bagaria, Brian D Rinker, Antonio J Forte

**Affiliations:** 1 Plastic Surgery, Robert D. and Patricia E. Kern Center for the Science of Health Care Delivery, Mayo Clinic Florida, Jacksonville, USA; 2 Health Science Research, Mayo Clinic Florida, Jacksonville, USA; 3 Surgery, Mayo Clinic Florida, Jacksonville, USA

**Keywords:** desmoplastic melanoma, malignant melanoma, tumor characteristics, ncdb

## Abstract

Objective

Desmoplastic melanoma (DM) is a rare variant of invasive malignancy of the skin pigmented cells. We present a comprehensive study reporting on US demographics, disease characteristics, and survival, to contribute to the current knowledge and raise awareness of this rare disease.

Materials and methods

The demographics of DM patients diagnosed from January 1, 2004, to December 31, 2015, were obtained by querying the National Cancer Database. The characteristics of DM were compared with common malignant melanoma (CMM) using univariate and multivariate regression models. Five-year overall survival (OS) curves were estimated using Kaplan-Meier analyses and the Cox proportional regression model.

Results

Our query found 5,895 patients diagnosed with DM and 292,939 patients with CMM. DM tended to present at an older age, a more advanced stage, and with a Breslow depth greater than 4 mm at diagnosis (P<.05). The Kaplan-Meier survival analysis demonstrated a five-year OS for DM and CMM of 75% and 76%, respectively, without any statistical difference (P=.07). Cox regression analysis demonstrated that age at diagnosis and comorbidities were independent predictors of five-year OS for DM (P<.001).

Conclusions

Older age, advanced stage, and higher Breslow depth were found to be independent positive factors associated with DM.

## Introduction

Desmoplastic melanoma (DM) is a rare variant of melanoma and is considered a type of spindle cell tumor with high amounts of collagen [1]. DM has an incidence of two per million, representing less than 4% of primary cutaneous melanoma diagnoses [2-3]. The presentation of this type of melanoma is unusual, and it is often difficult to detect [4]. DM may mimic benign and malignant neoplasms, presenting as an indurated discoid, plaque, or nodule, frequently without pigmentation [3]. Accordingly, the clinical diagnosis of this melanoma is challenging, resulting in infrequent misdiagnosis or underdiagnosis [5]. DM is located more frequently in the head and neck, followed by the extremities and trunk [6]. Surgical excision is the treatment of choice. However, given the described characteristics, these lesions are commonly detected when the depth of invasion is greater [5]. As a consequence, the resection of the tumor becomes more difficult, increasing the likelihood of wider resections that result in greater disfigurement at the surgical site. Due to the low incidence of the disease, few studies have described the characteristics of DM, and no study compared them with those of common malignant melanoma (CMM) [7-14]. The aim of this study was to perform a comprehensive national analysis of the characteristics and survival rate of DM and to compare them with those of CMM in the US.

## Materials and methods

This retrospective study utilized the National Cancer Database (NCDB) to identify DM cases registered between 2004 and 2015. The NCDB collects more than 70% of the new cancer cases in the US as part of the oncology hospital registry data. More than 34 million historical records are found in this database sponsored by the American College of Surgeons and the American Cancer Society [15]. The clinical data provided within the NCDB was compiled under conditions of de-identification, with stringent integrity revision for quality reliability.

Inclusion and exclusion criteria

**Figure 1:**
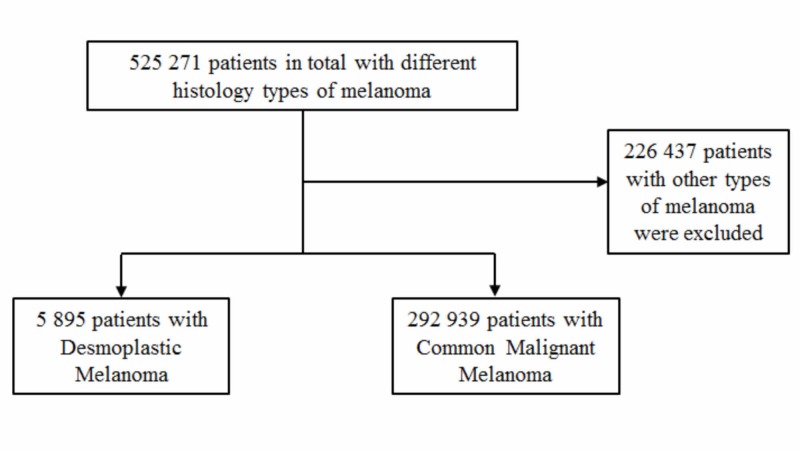
Inclusion and exclusion criteria

From 525,271 patients with all types of melanoma that were found in the NCDB database, we included only cases that were confirmed microscopically and classified as “desmoplastic melanoma” (n=5,895) and “malignant melanoma not otherwise specified” (n=292,939). “Malignant melanoma not otherwise specified” included patients with the histology type of melanoma not specified at diagnosis, considered common malignant melanoma (CMM) for the purpose of the study. Exclusion criteria included all the other histology types of melanoma different than DM and CMM (Figure 1).

Variables

The variables analyzed in this study are described in Table 1. Data abstracted from the database included demographic characteristics, such as age, sex, race, and year of diagnosis, and tumor characteristics, such as location, grade, and stage, Breslow depth, ulceration, surgery, radiation, regression, mitotic count, presence of lymph nodes positive, and brain, liver, and lung metastasis. For the multivariate regression analysis, tumor characteristics were considered independent variables except for the variable ‘grade,’ which was excluded due to the high number of cases with missing data. In addition, 4,284 DM and 265,187 CMM patients with missing data were excluded from the analysis. DM and CMM were considered our dependent variables of interest.

**Table 1:** Definition of variables

Variables	Definition
Demographics	
Age	Age at diagnosis
Sex	Male or female
Race	Race of the patient
Comorbidities	Charlson Comorbidity Score. Yes: ≥ 1 Charlson Comorbidity Score. No: 0 Charlson Comorbidity Score
Year at diagnosis	Year of initial diagnosis of the tumor, from 2004 to 2015
Characteristics	
Location	Location of the melanoma in the body
Grade	Described the melanoma’s resemblance to normal skin. Well-differentiated (grade I) was the most like normal tissue, and undifferentiated (grade IV) was the least like normal tissue
Stage	Reported pathologic stage group or clinical stage group, if the pathologic stage was not reported
Breslow depth	Thickness of the tumor's invasion
Ulceration	Absence of intact epidermis overlying the primary melanoma based upon histopathological examination
Radiation	Described if patients had any radiation therapy that was part of the first course of treatment
Regression	Primary tumor regression documented in the pathology report
Mitotic count	Number of mitotic figures found in one square millimeter (mm) surrounding either a "hot spot" with the most mitotic figures or a field with representative mitosis
Lymph nodes	Records the presence or not of positive regional lymph nodes examined by the pathologist and found to contain metastases
Brain metastasis	Brain metastasis at the time of diagnosis
Liver metastasis	Liver metastasis at the time of diagnosis
Lung metastasis	Lung metastasis at the time of diagnosis

Statistical analysis

Tumor characteristics were compared using chi-square. Univariate and multivariate logistic regression were performed to determine the risk of tumor characteristics in patients with DM as compared to CMM and adjusted for age and sex. Adjusted odds ratio (aOR) was found to compare the groups. A comparison of the five-year overall survival (OS) curves between DM and CMM was performed. The independent variables analyzed for five-year OS for DM included sex, age, comorbidities, stage, location, Breslow depth, mitosis, ulceration, radiation, and presence of lymph nodes positive. Cases with unspecified and missing data were excluded for the survival analysis. Survival time in months was determined from the date of diagnosis to the date of death, date last known to be alive, or December 2015. Survival curves were estimated using the Kaplan-Meier method and were compared using log-rank tests to determine the statistical significance between the survival curves for DM and CMM. The multivariable Cox proportional hazard model was used to calculate the hazard ratios of the overall survival (OS) for DM. SPSS, version 25 (SPSS, Inc., IBM, Chicago, Illinois, US) was used for the analysis and P<.05 was considered significant.

## Results

Demographic and tumor characteristics

**Figure 2:**
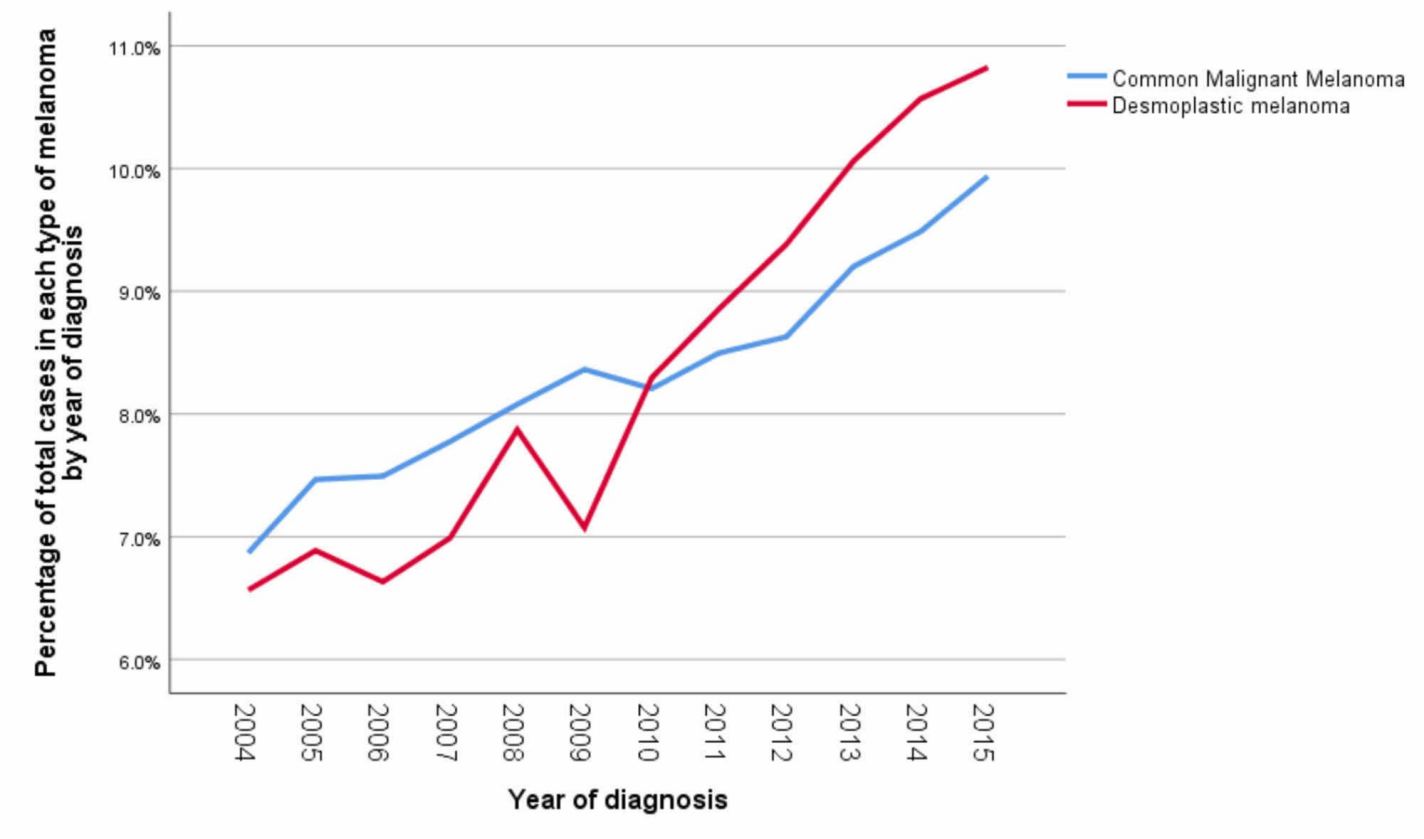
Comparison between percentages of Desmoplastic Melanoma and Common Malignant Melanoma by year of diagnosis

The percentage of DM patients was 1.1% (5,895) over all types of melanoma. We found that there has been a higher percentage of cases of DM over time since 2010 as compared to CMM (Figure 2). The mean age of patients with DM was 68.37 years; most were white (97.91%), males (67.53%), and between 61 and 80 years old (50.64%) (Table 2).

**Table 2:** Demographic characteristics of patients with Desmoplastic Melanoma and Common Malignant Melanoma

Demographic Characteristics	Desmoplastic Melanoma		Common Malignant Melanoma		P Value
	Total N=5,895		Total N=292, 939		
	n	%	n	%	
Age					
Mean (SD)	68.37 (14.09)	-	61.17 (16.09)	-	-
Median	70	-	62	-	-
Range	(7-90)	-	(0-90)	-	-
Age group					
<40 years	215	3.65	30,791	10.51	
40-60 years	1,414	23.99	102,465	34.98	
61-80 years	2,985	50.64	125,707	42.91	
>80 years	1,281	21.73	33,976	11.60	
Sex					
Male	3,981	67.53	165,749	56.58	
Female	1,914	32.47	127,190	43.42	
Race					0.18
White	5,772	97.91	284,369	97.07	
Black	43	0.73	1,821	0.62	
Other	36	0.61	2,308	0.79	
Unknown	44	0.75	4,441	1.52	
Year of diagnosis					
2004-2006	1,184	20.08	63,943	21.83	
2007-2009	1,293	21.93	70,941	24.22	
2010-2012	1,564	26.53	74,204	25.33	
2013-2015	1,854	31.45	83,851	28.62	

DM was more frequently found in the scalp and neck (24.17%), of grade III (0.64%) and stage II (55.15%), with a Breslow depth of more than 4 mm (35.44%), a mitotic count of 1 mitosis/mm^2^ or more (32.25%), negative lymph nodes (61.97%), and without any ulceration (78.56%) or regression (42.27%). On the other hand, CMM was more frequently located in the trunk (28.25%), of grade III (0.54%) and stage I (36.12%), with a Breslow depth of less than or equal to 1 mm (35.14%), negative lymph nodes (27.41%), a mitotic count of 1 mitosis/mm^2^ or more (13.10%), and without any ulceration (71.06%) or regression (28.84%) (Table 3).

**Table 3:** Tumor characteristics of patients with Desmoplastic Melanoma and Common Malignant Melanoma

	Desmoplastic Melanoma		Common Malignant Melanoma		P Value
	Total N=5,895		Total N=292,939		
	n	%	n	%	
Location					
Lips	87	1.48	722	0.25	
Eyelid	29	0.49	1,485	0.51	
External ear	182	3.09	8,875	3.03	
Other parts of face, site unspecified	1,447	24.55	35,209	12.02	
Scalp and neck	1,425	24.17	22,976	7.84	
Trunk	1,024	17.37	82,764	28.25	
Upper extremities and shoulder	1,314	22.29	68,082	23.24	
Lower extremities and hip	297	5.04	50,403	17.21	
Overlapping lesion of skin	14	0.24	350	0.12	
Skin, site unspecified	76	1.29	22,073	7.54	
Grade					.002
Grade I	9	0.15	1,083	0.37	
Grade II	8	0.14	788	0.27	
Grade III	38	0.64	1,585	0.54	
Grade IV	14	0.24	490	0.17	
Cell type not determined	5,826	98.83	288,993	98.65	
Stage					
Stage 0	40	0.68	90,983	31.06	
Stage I	1,576	26.73	105,810	36.12	
Stage II	3,251	55.15	27,247	9.3	
Stage III	390	6.62	20,571	7.02	
Stage IV	156	2.65	18,499	6.31	
Unknown	482	8.18	29,829	10.18	
Breslow depth					
≤1 mm	1,051	17.83	102,950	35.14	
1.01- 2 mm	1,079	18.3	32,632	11.14	
2.01-4mm	1,331	22.58	18,310	6.25	
>4 mm	2,089	35.44	12,362	4.22	
Unknown	345	5.85	126,685	43.25	
Ulceration					
No	4,631	78.56	211,096	72.06	
Yes	862	14.62	28,347	9.68	
Unknown	402	6.82	53,493	18.26	
Surgery					
No, diagnosed at autopsy	92	1.56	22,890	7.81	
Yes	5,798	98.35	268,861	91.78	
Unknown	5	0.08	1,188	0.41	
Radiation					
No	4,964	84.21	278,907	95.21	
Yes	899	15.25	11,865	4.05	
Unknown	32	0.54	2,167	0.74	
Regression					
No	2,492	42.27	84,497	28.84	
Yes	132	2.24	10,143	3.46	
Unknown	3,219	54.61	194,816	66.5	
Mitotic count					
No presence of mitosis	733	12.43	35,897	12.25	
<1 mitosis/mm^2^	239	4.05	7,293	2.49	
≥1 mitosis/mm^2^	1,901	32.25	38,376	13.10	
Unknown	2,970	50.38	207,891	70.97	
Lymph nodes					
Negative	3,653	61.97	80,296	27.41	
Positive	339	5.75	23,428	8	
Unknown	1,903	32.28	189,215	64.59	
Brain metastasis					
No	3,376	57.27	150,251	51.29	
Yes	8	0.14	3,391	1.16	
Unknown	2,511	42.6	139,297	47.55	
Liver metastasis					
No	3,374	57.23	151,499	51.72	
Yes	10	0.17	2,077	0.71	
Unknown	2,511	42.6	139,363	47.57	
Lung metastasis					
No	3,338	56.62	149,532	51.05	
Yes	46	0.78	4,049	1.38	
Unknown	2,511	42.6	139,358	47.57	

Regarding therapy, most of the DM (98.35%) and CMM (91.78%) patients underwent surgery. Furthermore, most of DM (84.21%) and CMM (95.21%) patients did not receive radiation to their lesions. With respect to metastasis, most of the cases in both types of melanoma did not have metastasis at diagnosis, although for DM patients, the most common metastasis, when it was found, was lungs (0.78%), followed by liver (0.17%), and brain (0.14%) metastasis, respectively (Table 3).

Tumor characteristics comparison

**Figure 3:**
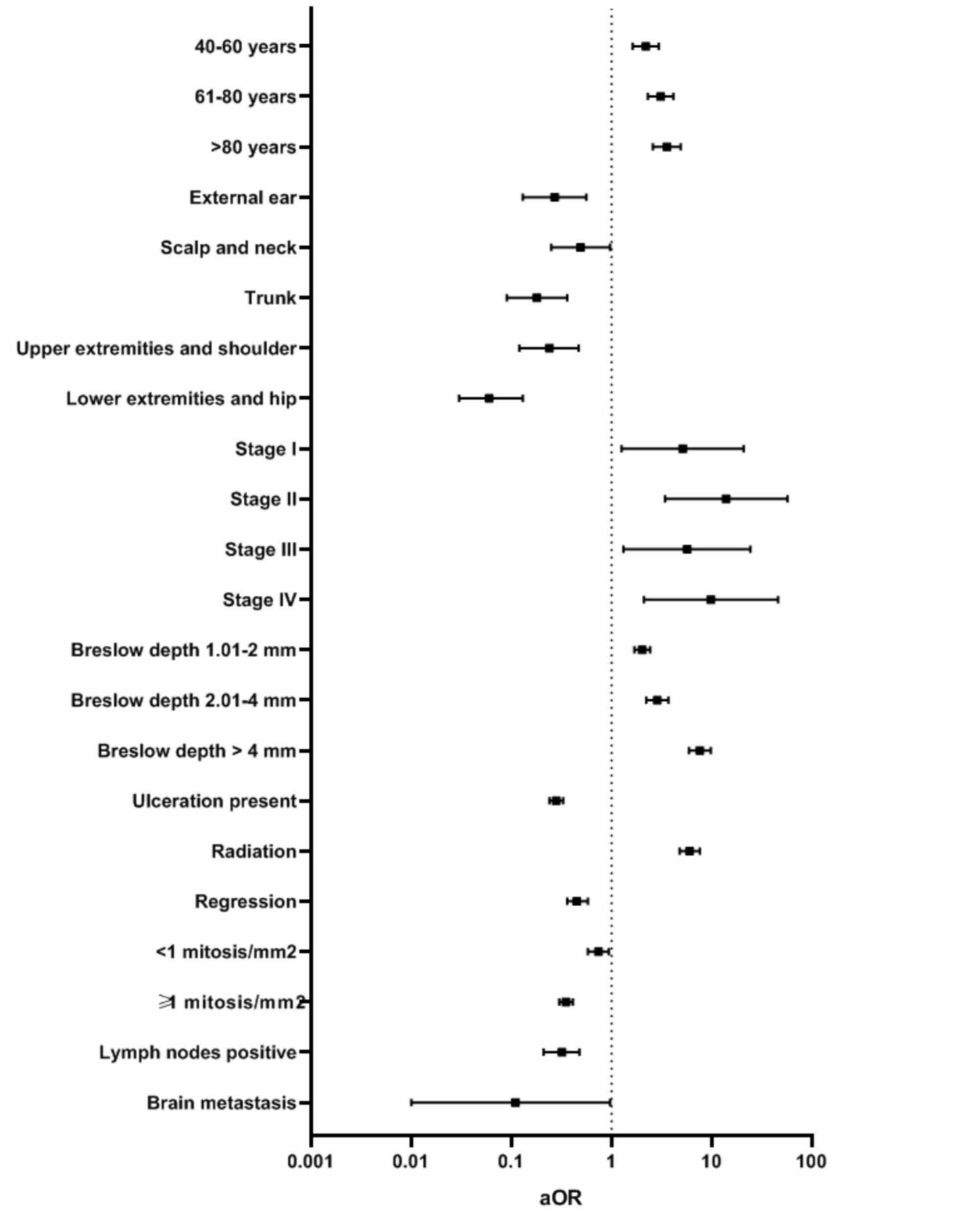
Adjusted odds ratio (aOR) with 95% confidence interval for tumor characteristics of patients with Desmoplastic Melanoma compared to Common Malignant Melanoma

One-thousand six-hundred and eleven cases with DM and 27,752 cases with CMM were analyzed to compare tumor characteristics. We found that DM was more likely to be diagnosed in patients older than 80 years (adjusted odds ratio (aOR), 3.57; 95% confidence interval (CI), 2.59-4.91; P<.001), stage IV (aOR, 9.85; 95% CI, 2.11-46.00; P=.004), with a Breslow depth of greater than 4 mm (aOR,7.63; 95% CI, 5.95-9.80; P<.001); and be treated with radiation (aOR, 6.03; 95% CI, 4.78-7.62; P<.001), as compared to CMM. On the other hand, DM was less likely to present in the lower extremities and hip (aOR, 0.06; 95%CI, 0.003-0.13; P<.001), have ulceration (aOR, 0.28; 95% CI, 0.24-0.33; P<.001), present regression (aOR, 0.45; 95% CI, 0.35-0.58; P<.001), have a mitotic count of 1 mitosis/mm^2^ or more (aOR, 0.35; 95% CI, 0.30-0.41; P<.001), present lymph node involvement (aOR, 0.32; 95% CI, 0.21-0.48; P<.001), and metastasis to the brain at diagnosis (aOR, 0.11; 95% CI, 0.01-0.97; P=.047), as compared to CMM (Table 4, Figure 3). We did not find a statistical difference between DM and CMM regarding sex, surgery, and metastasis to the liver and lungs.

**Table 4:** Univariate and multivariate analysis of factors associated with Desmoplastic Melanoma Abbreviations: OR, odds ratio; aORs, adjusted odds ratios *Reference value **Only aORs associated with a CI not crossing 1.0 are shown

	Univariate Analysis			Multivariate Analysis		
Variables	OR	95% CI	P Value	aORs**	95% CI	P Value
Age						
< 40 years	1.00*	-	-	1.00*	-	-
40-60 years	2.31	1.74-3.07		2.2	1.63-2.97	
61-80 years	4.5	3.43-5.91		3.09	2.31-4.14	
>80 years	6.27	4.67-8.41		3.57	2.59-4.91	
Sex						
Male	1.00*	-	-	1.00*	-	-
Female	0.63	0.56-0.70		-	-	-
Location						
Lip	1.00*	-	-	1.00*	-	-
Eyelid	0.16	0.04-0.56	.005	-	-	-
External ear	0.16	0.09-0.28		0.27	0.13-0.56	
Other parts of face, site unspecified	0.37	0.22-0.64		-	-	-
Scalp and neck	0.38	0.22-0.65		0.49	0.25-0.97	.04
Trunk	0.08	0.05-0.14		0.18	0.09-0.36	
Upper extremities and shoulder	0.12	0.07-0.21		0.24	0.12-0.47	
Lower extremities and hip	0.03	0.01-0.05		0.06	0.03-0.13	
Stage						
Stage 0	1.00*	-	-	1.00*	-	-
Stage I	4.9	1.57-15.31	.006	5.14	1.26-20.92	.02
Stage II	24.83	7.97-77.39		13.99	3.42-57.28	
Stage III	3.92	1.24-12.38	.02	5.69	1.32-24.49	.02
Stage IV	11.74	3.46-39.83		9.85	2.11-46.00	.004
Breslow depth						
≤1 mm	1.00*	-	-	1.00*	-	-
1.01-2 mm	1.72	1.44-2.04		2.03	1.69-2.43	
2.01-4 mm	4.07	3.44-4.81		2.86	2.22-3.69	
>4 mm	9.35	7.98-10.96		7.63	5.95-9.80	
Ulceration						
No	1.00*	-	-	1.00*	-	-
Yes	0.55	0.48-0.63		0.28	0.24-0.33	
Surgery						
No, diagnosed at autopsy	1.00*	-	-	1.00*	-	-
Yes	1.23	0.3-5.09	.77	-	-	-
Radiation						
No	1.00*	-	-	1.00*	-	-
Yes	7.81	6.65-9.19		6.03	4.78-7.62	
Regression						
No	1.00*	-	-	1.00*	-	-
Yes	0.38	0.30-0.48		0.45	0.36-0.58	
Mitotic count						
No presence of mitosis	1.00*	-	-	1.00*	-	-
<1 mitosis/mm^2^	0.84	0.69-1.04	.11	0.74	0.58-0.94	.01
≥1 mitosis/mm^2^	0.65	0.57-0.73		0.35	0.3-0.41	
Lymph nodes						
Negative	1.00*	-	-	1.00*	-	-
Positive	0.31	0.26-0.38		0.32	0.21-0.48	
Brain metastasis						
No	1.00*	-	-	1.00*	-	-
Yes	0.46	0.06-3.34	.44	0.11	0.01-0.97	.047
Liver metastasis						
No	1.00*	-	-	1.00*	-	-
Yes	0.74	0.18-3.06	.68	-	-	-
Lung metastasis						
No	1.00*	-	-	1.00*	-	-
Yes	1.86	0.97-3.58	.06	-	-	-

Survival analysis

**Figure 4:**
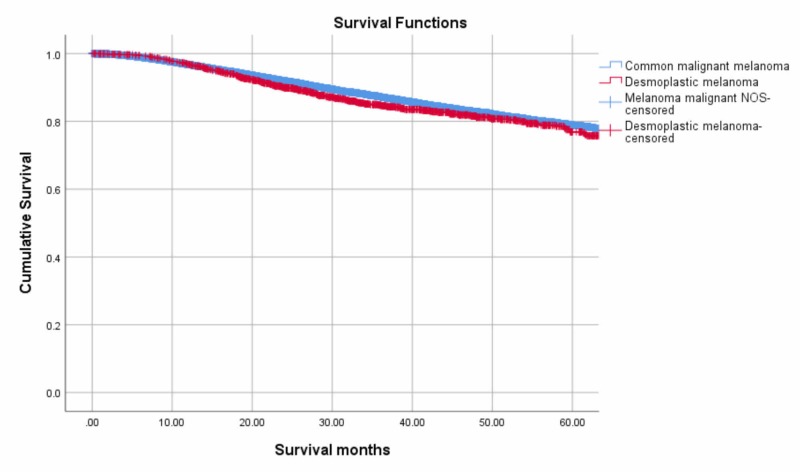
Kaplan-Meier curves depicting five-year overall survival of patients with Desmoplastic Melanoma and Common Malignant Melanoma

Patients with DM had a five-year OS rate of 75 %, whereas patients with CMM had a five-year OS rate of 76 %. Figure 4 demonstrates the Kaplan Meier survival curves (log rank, P<.07). The multivariable Cox proportional model revealed that age at diagnosis (hazard ratio (HR), 1.06; 95% CI, 1.04-1.07; P<.001) and presence of comorbidities (HR, 2.08; 95% CI, 1.56-2.77; P<.001) were independent positive predictors of OS in patients with DM while the location of the DM in the upper extremities and shoulder was found to reduce the risk of the overall death in these patients (HR, 0.61; 95% CI, 0.43-0.87; P=0.01) (Table 5).

**Table 5:** Cox proportional hazard regression analysis of prognostic factors for Overall Survival in patients with Desmoplastic Melanoma Abbreviations: HR, hazard ratio *Reference value

	Desmoplastic Melanoma		
Characteristic		HR (95% CI)	P Value
Age	1.06 (1.04-1.07)		
Sex			
Male	1.00*		
Female	0.73 (0.52-1.01)		.06
Comorbidities			
No	1.00*		
Yes	2.08 (1.56-2.77)		
Location			
Head and neck (lips, eyelid, external ear, other parts of face, site unspecified, and scalp and neck)	1.00*		
Trunk	0.75 (0.52-1.07)		.11
Upper extremities and shoulder	0.61 (0.43-0.87)		.01
Lower extremities and hip	0.78 (0.37-1.64)		.51
Breslow depth			
≤1 mm	1.00*		
1.01-2 mm	1.22 (0.70 -2.12)		.48
2.01-4 mm	0.80 (0.44-1.47)		.48
>4 mm	1.52 (0.88-2.63)		.14
Stage			
Stage 0	1.00*		
Stage I	0.20 (0.03-1.61)		.13
Stage II	0.32 (0.04-2.35)		.26
Stage III	0.39 (0.05-3.13)		.37
Stage IV	0.88 (0.11-7.20)		.91
Lymph nodes			
Negative	1.00*		
Positive	1.94 (0.97-3.89)		.06
Ulceration			
No	1.00*		
Yes	1.12 (0.81-1.55)		.49
Radiation			
No	1.00*		
Yes	0.71 (0.50-1.02)		.06
Mitotic count			
No presence of mitosis	1.00*		
<1 mitosis/mm^2^	1.05 (0.58-1.91)		.87
≥1 mitosis/mm^2^	1.29 (0.90-1.83)		.16

## Discussion

DM is considered an uncommon subtype melanoma with a frequency of 1% to 4% [14]. In our study, we found that DM corresponded to 1.1% of the total types of melanoma reported on the NCDB. This low frequency of DM is concerning, as this could be related to a misdiagnosis of the disease; however, the increase in the percentage of cases reported over the years may indicate a decreasing trend in misdiagnosis. It may also be a result of the efforts for early detection and the increase in total cases of melanoma [16]. The female-to-male ratio found in this study was approximately 1:2, as it was previously found by Xu et al. [17]. Concerning the demographic characteristics, most of the patients with DM were white men over the age of 80, as is typically found in patients with CMM [18].

In our study, the most frequent locations of DM were the head and neck, specifically the scalp and neck, followed by the upper limbs, shoulders, and trunk. This differs from our findings regarding CMM, which was most often found in the trunk, followed by the face and the upper limbs and shoulders. These differences may be linked to a change in clothing, lifestyle habits, and chronic ultraviolet exposure independent of the type of melanoma [19]. DM is often located in places that are not easily seen, which might be an additional factor associated with delayed diagnosis. Furthermore, DM is also challenging to diagnose due to the difficulty to recognize the melanoma-associated patterns [20], its variable appearance, and the absence of pigmentation [4,21].

Our study showed that DM patients presented at diagnosis with higher grades, advanced stage, and deeper Breslow depth (≥4 mm) when compared with patients affected by CMM. Moreover, radiation therapy was more likely to be given to patients with DM, probably due to the delay in diagnosis and its high propensity to recur, as this type of treatment is reserved for unresectable cases, and to prevent the recurrence of the disease [10,19].

Associations of high mitotic rates with the presence of positive sentinel lymph nodes, higher rates of recurrence, and lower survival rates have been demonstrated in CMM [22-23]. In our study, we found that DM patients had less risk to have an increased count of mitosis compared to CMM, suggesting that DM has a better prognosis than CMM. Other predictors of prognosis previously described are the presence of ulceration and sentinel lymph nodes [24]. The presence of ulceration favors the dissemination of the tumor after modifying the local environment of the DM in the skin and directly impacts the rates of survival [22,25]. In our study, DM lesions were less likely to have ulceration as compared to CMM and to influence the death rates of DM patients.

Currently, regression and its relationship with prognosis are controversial. Some studies suggest that regression is an indicator of poor prognosis [26] while others propose that it is related to better outcomes [27]. Our study showed that regression is more likely to occur in CMM than in DM.

Sentinel lymph node biopsy allows the detection of micrometastasis in the regional lymph nodes with high sensitivity and specificity and is also a prognostic factor of survival [23]. In the present study, DM patients had less risk of lymph node involvement as compared to CMM patients. This finding supports the idea that DM is related to fewer metastases to lymph nodes (rate of 0%-15%) as previously described by Andreevscaia et al. [4]. In addition, Sims et al. found that metastases to regional lymph nodes were less common to appear in DM of the head and neck as compared to CMM [28].

Systemic metastases are not frequent in DM, affecting the lung, liver, and bone in 7% to 44% of patients [10]. In our study, we found that the most frequent metastasis was the lung (0.78%), followed by the liver (0.17%) and brain (0.14%). Probably, the rates of metastases were overestimated due to the lower number of DM cases reported in the literature compared with the total number of DM cases described in our study.

In the survival analysis, the five-year OS in patients with DM was 75%, without any statistical difference with CMM. Previously, Khan et al. found a five-year OS of 79.5% in patients with DM of the head and neck, diagnosed from 1992 to 2013 [3]. The difference in these OS rates may be due to the high number of cases presented in our analysis. In our analysis, the DM survival rate was determined by the advanced age and the presence of comorbidities in the patients analyzed.

Known prognostic factors like Breslow depth, ulceration, mitosis, and lymph nodes involvement were not associated to increase the risk of death, probably due to the survival being related to other causes of death, and not specifically to DM [29].

There are some important limitations to this study related to the source of data from the NCDB. In particular, our results were dependent on the information compiled in the database, which was not always complete. In an effort to obtain the most accurate results possible, we excluded missing information that could affect the models. Furthermore, as all the database information is provider dependent, some cases of DM could have been misclassified. For this reason, we considered the variable ‘histology’ to define the DM and CMM cases confirmed by the pathologist. Regarding the survival curves, we were unable to address the disease-specific survival because the NCDB did not allow us to obtain this information. However, the OS gave us an approximation of the survival of these DM patients. Despite these limitations, we believe this study reports a valuable analysis of the demographics and tumor characteristics of DM patients. Moreover, the comparison of DM with CMM gives us an orientation of the aggressiveness of the disease and underscores the importance of an early diagnosis. However, further prospective studies are needed to better explain the progression and biology of DM.

Our findings support the idea that DM continues being misdiagnosed or diagnosed at a more advanced stage of the disease despite the increasing rate of diagnosis over time. Older age, advanced stage, higher Breslow depth, and radiation therapy were more likely to be present in DM patients as compared to CMM. This study gives significant insights into this very rare type of melanoma. Surgeons should be aware of DM and its characteristics in order to control the burden of the disease and to improve management strategies in the future.

## Conclusions

Our study suggests that DM probably continues being diagnosed at a more advanced stage of the disease, despite its prevalence over time. Older age, advanced stage, higher Breslow depth, and radiation therapy were more likely to be present in DM patients as compared to CMM. This study gives significant insights into this very rare type of melanoma. Surgeons should be aware of DM and its characteristics in order to control the burden of the disease and to improve management strategies in the future.
